# Acute Effects of Pelemir-Enriched Bread on Postprandial Glucose and Hormonal Responses in Adults with Obesity, Type 2 Diabetes, and Normal Weight: A Two-Phase Exploratory Study

**DOI:** 10.3390/nu17172819

**Published:** 2025-08-29

**Authors:** Ozlem Soyluk Selcukbiricik, Fulya Calikoglu, Cemile Idiz, Gulay Dura, Gokmen Sir, Onder Yuksel Eryigit, Isik Kulaksiz, Mustafa Hakan Yilmazturk, Ayse Kubat Uzum, Kubilay Karsidag, Ilhan Satman

**Affiliations:** 1 Division of Endocrinology and Metabolism, Department of Internal Medicine, Istanbul Faculty of Medicine, Istanbul University, Istanbul 34093, Türkiye; bfulyacalikoglu@gmail.com (F.C.); cemileidiz@gmail.com (C.I.); akubatuzum@gmail.com (A.K.U.); kubilaykarsidag@yahoo.com (K.K.); satmandiabet@gmail.com (I.S.); 2 Medical Laboratory, Istanbul Metropolitan Municipality, Istanbul 34440, Türkiye; gulaydura@hotmail.com (G.D.); isikkulaksiz@gmail.com (I.K.); 3 Istanbul Metropolitan Municipality, Istanbul 34440, Türkiye; gokmensir@hotmail.com (G.S.); onder.eryigit@gmail.com (O.Y.E.); yilmazturkhakan@yahoo.com (M.H.Y.)

**Keywords:** *Cephalaria syriaca*, pelemir, postprandial metabolism, GLP-1, insulin, incretin, functional bread, polyphenols

## Abstract

**Background:** Pelemir (*Cephalaria syriaca*) is a bitter-tasting ancestral legume with a high polyphenol content and emerging potential as a functional food ingredient. This study investigated the acute metabolic effects of pelemir-enriched bread in adults. **Methods:** In this two-phase non-randomized trial, 60 participants in three groups (*n* = 20 per group: healthy controls [HCs], individuals with obesity [OB], and individuals with type 2 diabetes [T2D]) consumed regular or pelemir-enriched bread on two separate test days. Postprandial glucose, insulin, C-peptide, GLP-1, PYY, ghrelin, leptin, triglyceride, and IL-6 were measured over 120 min. Subjective appetite ratings were evaluated using visual analog scales (VASs). The incremental area under the curve (iAUC) values were compared using Wilcoxon tests and linear mixed-effects models. **Results:** Pelemir-enriched bread significantly increased iAUCs for insulin (*p* = 0.014), C-peptide (*p* = 0.046), and GLP-1 (*p* = 0.039) compared to regular bread. There was no significant change in iAUC for glucose. Group-stratified analyses showed a higher postprandial iAUC of glucose, insulin, and C-peptide in the OB group compared to the HC group. VAS-based appetite ratings did not show significant changes in hunger, fullness, or desire to eat, but a borderline significant reduction was observed in prospective food consumption after pelemir-enriched bread (*p* = 0.050). **Conclusions**: Acute consumption of pelemir-enriched bread may modulate postprandial insulin and incretin responses. Its modest impact on subjective appetite regulation supports further investigation of pelemir as a functional food rich in polyphenols, especially in populations with metabolic dysfunction.

## 1. Introduction

Obesity and type 2 diabetes (T2D) are among the most prevalent and burdensome noncommunicable diseases worldwide, driven primarily by excessive energy intake, sedentary lifestyle, and environmental changes apart from genetics. Globally and in Türkiye, the rise in these conditions has led to significant public health challenges, prompting urgent calls for preventive and therapeutic strategies targeting modifiable lifestyle factors, particularly diet [1,2].

Generally, dietary approaches have received increasing attention, including consumption of foods rich in polyphenols and fibers to improve metabolic control, support weight management, and reduce postprandial glucose fluctuations [3,4,5,6]. Bread is the main source of carbohydrates in the traditional Turkish diet, with an average daily consumption exceeding 270 g/day, significantly higher than the global average [2]. Although white bread still dominates the market (~85%), the demand for alternative flours and functional bread formulations has been steadily increasing [2,7].

Pelemir (*Cephalaria syriaca* L.), a native flowering plant of the Caprifoliaceae family, has traditionally been used in Anatolia to improve dough rheology and extend bread shelf life [8,9]. Contrary to previous misclassifications as a weed, this cold-resistant and drought-tolerant plant is now recognized for its nutritional and pharmacological potential. Although proteins and lipids are more abundant in seeds, bioactive phenolic compounds have been reported to be found more in flowers as well as in shoots, seeds, and roots [10]. Its seeds contain higher amounts of dietary fiber (9–30%), protein (14–21%), and polyunsaturated fats, including linoleic and oleic acids, than its flowers [11,12,13]. Furthermore, Cephalaria species are rich in bioactive compounds such as triterpenes, polyphenols, and flavonoids with antioxidant, anti-inflammatory, and antidiabetic properties [14,15,16].

In vitro studies have shown that Cephalaria extracts exert α-glucosidase and α-amylase inhibition, suggesting a potential for postprandial glucose modulation [17]. However, despite promising biochemical and pharmacognostic evidence, there are no published human clinical studies that evaluate the effects of *Cephalaria syriaca* consumption on postprandial glucose metabolism, gut hormone responses, or appetite regulation.

Bread produced with pelemir-added flour—currently marketed in Türkiye under the brand name “Akdeniz Bread^®^”—has a similar carbohydrate content to white bread but differs in fat, protein, and polyphenol levels. These differences may affect postprandial endocrine responses and glycemic profiles. However, the seed-based composition, as distinct from the flower or root extracts used in previous studies, poses unique metabolic implications that warrant clinical evaluation.

We hypothesized that pelemir-added bread would attenuate the postprandial glycemic response and enhance the release of incretin and satiety hormones compared to standard (regular) white bread, particularly in metabolically affected individuals. Therefore, the present three-arm, parallel-group, two-phase study was designed to investigate the acute effects of pelemir-added bread on glucose metabolism, gastrointestinal hormones, and appetite-related parameters in healthy individuals, patients with obesity, and patients with T2D. This is the first human study to evaluate *Cephalaria syriaca* in a real-food matrix and across different metabolic phenotypes.

## 2. Materials and Methods

This non-randomized, two-phase study was conducted at Istanbul University, Istanbul Medical Faculty, Department of Internal Medicine, Division of Endocrinology and Metabolism, between April 2022 and February 2023. The study was approved by the Institutional Review Board (05.04.2022-834721) and registered on ClinicalTrials.gov (NCT05687812). All participants provided their written informed consent. The study adhered to the principles outlined in the Declaration of Helsinki.

### 2.1. Study Population

Participants were stratified into three groups (*n* = 20 each): healthy controls (HCs), individuals with obesity (OB), and patients with type 2 diabetes (T2D). Inclusion criteria for HCs were age >18 years, body mass index (BMI) between 18.5 and 24.9 kg/m^2^, having no chronic diseases, no regular use of medication or supplements, non-smoking status, and confirmed with normal glucose tolerance by a standard 2 h oral glucose tolerance test. The OB group included individuals with a BMI of ≥30 kg/m^2^ who did not have diabetes. The T2D group consisted of individuals diagnosed with T2D mellitus using oral antidiabetic medications without insulin, dipeptidyl peptidase-4 inhibitors, or glucagon-like peptide-1 (GLP-1) receptor agonists.

Exclusion criteria for all participants included pregnancy, breastfeeding, major systemic diseases (e.g., renal diseases, pulmonary diseases, malignancy), excessive physical activity (more than 3 h per week of vigorous exercise), and current use of nutraceuticals. Physical activity levels were assessed using the International Physical Activity Questionnaire-short form, which has been validated for Turkish adults.

### 2.2. Study Design and Interventions

Participants completed two study visits in a fixed sequence (regular bread followed by pelemir bread) separated by a one-week interval. Because the order was not randomized and no counterbalancing was used, this should be considered a sequential two-phase design rather than a crossover trial. The one-week interval was logistical, rather than a pharmacological washout, and carry-over/period effects cannot be excluded. Participants with T2D did not take their medications (metformin, sulfonylureas, or sodium–glucose linked transporter-2 inhibitors) on the morning of the test day. No medications were stopped before the test day. This decision was made to ensure patient safety and to capture real-life glycemic responses. We acknowledge that continued therapy may contribute to lower baseline values in the T2D group compared to the OB group; however, our primary outcome was the within-subject difference between bread types, which is unaffected by between-group baseline differences.

Each test meal contained 50 g of available carbohydrates of any bread type and was consumed in 10 min with 250 mL of water after an overnight fast (10–12 h). Istanbul Halk Ekmek Co. (Istanbul, Türkiye) produced the breads, and the pelemir (*Cephalaria syriaca* L.) flour was provided by Ziya Organik Tarım İşletmeleri Co. (Istanbul, Türkiye). The test bread included 0.3% pelemir flour (*w*/*w*). Although the pelemir concentration was relatively low, this amount was selected on the basis of preproduction trials and sensory limitations. According to food technologists at Istanbul Halk Ekmek Co., higher concentrations of pelemir (0.5%) led to a strong bitter taste, adversely affecting palatability and limiting acceptability in routine consumption. Therefore, 0.3% was identified as the optimal dose of the formulation. At this level, no significant impact is expected on the macronutrient profile—especially protein or fiber. Therefore, the inclusion of pelemir was primarily intended for its polyphenol-rich bioactivity, rather than for its contribution to bulk nutrient content.

The nutritional composition and ingredients for the breads are presented in Table 1. Calculating the available carbohydrates accounted for the fiber content.

### 2.3. Anthropometry and Body Composition

Height was measured using a wall-mounted stadiometer, and weight and body composition (including fat mass and fat-free mass) were evaluated using a segmental bioimpedance analyzer (TANITA BC 420 MA). BMI was calculated as weight (in kilograms) divided by height (in meters squared).

### 2.4. Biochemical Measurements

Venous blood samples were collected just before (0 min) and 30, 60, 90, and 120 min after bread ingestion via an indwelling cannula placed in the antecubital vein. The 0 min sample was a fasting measurement, obtained prior to bread consumption. Blood samples were analyzed within 30 min of collection in a certified centralized laboratory.

The following methods were used:

- Glucose: Hexokinase/G-6-PDH (Abbott CI 8200);
- Triglycerides: Glycerol phosphate oxidase;
- Insulin and C-peptide: Chemiluminescent microparticle immunoassay (CMIA);
- GLP-1, peptide YY (PYY), ghrelin, leptin, and interleukin-6 (IL-6): Enzyme-linked immunosorbent assay (ELISA) kits (Elabscience; Houston, TX, USA).

The intra- and inter-assay coefficients of variation (c.v.) for all ELISA tests were <10% and <15%, respectively, according to the manufacturer’s instructions.

### 2.5. Appetite Assessment

Subjective sensations of hunger, fullness, desire to eat, and prospective food consumption were evaluated using a 100 mm visual analog scale (VAS), with two endpoints labeled “not at all” and “extremely”. Participants rated their feelings at the beginning of the study (0 min) and 30, 60, 90, and 120 min after eating each test bread (pelemir-enriched or regular). VAS outcomes were included as secondary endpoints to assess the short-term subjective satiety and appetite-modulating potential of pelemir-enriched bread.

### 2.6. Outcome Measures

The primary outcome was the incremental area under the curve (iAUC) for postprandial glucose (mg.min/dL). Secondary outcomes included iAUCs for insulin, C-peptide, GLP-1, PYY, leptin, ghrelin, triglycerides, IL-6, and all VAS parameters. The iAUCs were calculated using the trapezoidal rule with baseline subtraction.

### 2.7. Sample Size Calculation

Before enrollment, a power analysis was performed using the G*Power software (v3.1.9.4). Assuming a paired sample design, a size of effect within the subject (Cohen’s d) of 0.7, an α level of 0.05, and a power of 80%, a minimum of 19 participants per group was required to detect significant differences in iAUC for postprandial glucose. To account for secondary endpoints and possible variability, 20 participants were included in each group (total *n* = 60).

### 2.8. Statistical Analysis

Statistical analysis was performed using SPSS v21. The normal distribution of data was assessed by histogram inspection and Shapiro–Wilk tests.

Normally distributed data were presented as mean ± standard deviation (SD). Data that did not follow the normal distribution were presented as median and interquartile range (IQR) and analyzed using nonparametric tests. As the GLP-1 level shows a right-hand distribution and can be close to the lowest detection limit in certain individuals, all values are checked for detectability, and appropriate nonparametric tests were applied. Outliers were carefully evaluated, and visualization techniques were selected accordingly. Within-subject comparisons of bread types were evaluated using paired t-tests or Wilcoxon signed-rank tests, depending on distribution. Comparisons between groups were made using the Kruskal–Wallis or Mann–Whitney U tests.

To explore the effect of bread type while accounting for participant clustering and group differences, linear mixed effects models (LMMs) were constructed using bread type as a fixed factor, participant’s ID as a random intercept, and group (HC, OB, T2D) as a covariate. Bread × group interaction terms were included when appropriate. Although nonparametric tests were used to compare within the group, LMMs were used to estimate the overall effects of the bread type throughout the cohort. This approach adjusts for between-group differences and estimates marginal mean differences across the entire cohort, which may differ in magnitude from unadjusted within-group medians. Importantly, this study involved two repeated measures per participant in a fixed order; LMMs were used to account for subject-level clustering.

For each VAS outcome, iAUC was calculated using the trapezoidal method to reflect the overall postprandial response. Data were analyzed using Wilcoxon signed-rank tests to compare responses between the two breads. The results are presented as median [IQR].

All tests were two-tailed, and *p* < 0.05 was considered statistically significant. Trends (0.05 < *p* < 0.10) are also reported descriptively and interpreted cautiously without inferential claims.

## 3. Results

### 3.1. Characteristics of the Participants

A total of 60 participants (*n* = 20 per group: HC, OB, T2D) completed the study. Demographic and body composition characteristics are presented in Table 2.

The median age was significantly higher in the T2D group (52.7 [46.0–59.0] years) compared to the OB (39.5 [33.5–44.3]) and HC (38.0 [32.0–45.8]) groups (*p* < 0.001). BMI was also significantly elevated in OB (35.1 [31.5–38.2] kg/m^2^) and T2D (31.5 [25.8–37.6]) groups compared to the HC group (22.5 [20.9–23.7]) (*p* < 0.001). The differences in body weight and fat mass were similar to the categorization of the group. No significant differences between groups were observed in height (*p* = 0.084).

### 3.2. Fasting Biochemical Values

The biochemical parameters of fasting (0 min) measured immediately before bread consumption are shown in Table 3. There were no significant differences in fasting glucose, insulin, PYY, or ghrelin levels between test days (pelemir-enriched vs. regular bread; *p* > 0.05 for all).

Fasting C-peptide levels showed a marginal trend toward higher values on the day of pelemir-enriched bread consumption (median: 2.1 [0.7–7.1] ng/mL vs. 2.0 [0.6–4.1] ng/mL; *p* = 0.051). Similarly, fasting leptin levels appeared to be lower on the day of pelemir-enriched bread consumption (1005.0 [53.0–5251.0] pg/mL) than with regular bread (1347.0 [66.0–5877.0] pg/mL), although this did not reach statistical significance (*p* = 0.063). These findings do not suggest significant differences in baseline biochemical status between test days.

### 3.3. Incremental Postprandial Responses

The iAUC values for glucose and hormonal responses over 120 min after bread consumption are presented in Table 4.

No significant differences in iAUC for glucose were observed between pelemir-enriched and regular bread in any of the metabolic groups (*p* > 0.05). For insulin and C-peptide, although no within-group comparisons reached statistical significance in the HC or T2D groups, a modest but significant decrease in the iAUC for C-peptide was observed in the OB group after pelemir-enriched bread consumption (median: 10.8 [6.0–24.8] vs. 11.1 [5.1–19.5] ng.min/mL; *p* = 0.05).

The iAUC for GLP-1 increased in the HC group with pelemir-enriched bread (median: 414.6 [14.6–1801.6] pg.min/mL) compared to regular bread (197.7 [7.4–994.1]; *p* = 0.022), while no significant differences were observed in the OB or T2D groups. For other hormones and inflammatory markers (PYY, leptin, IL-6, and ghrelin), no statistically significant differences were found between groups.

### 3.4. Effect of Bread Type on Incremental Areas Under the Curve for Postprandial Responses (Group-Adjusted Analysis)

Linear mixed-effects models (LMMs) adjusted for group (HC, OB, T2D) revealed significant effects of bread type on several postprandial iAUC parameters (Table 5). Compared to regular bread, pelemir-enriched bread resulted in significantly higher iAUC values for insulin (β = +555.6, 95% CI 110.2 to 1001.0, *p* = 0.014), C-peptide (β = +35.9, 95% CI 0.7 to 71.1, *p* = 0.046), and GLP-1 (β = +1234.8, 95% CI 60.3 to 2409.2, *p* = 0.039).

No statistically significant differences were observed in iAUC for glucose, leptin, IL-6, ghrelin, or PYY values between the two bread types in all groups.

### 3.5. Between-Group Differences on Incremental Areas Under the Curve for Postprandial Responses

Comparisons between groups were performed using LMMs to evaluate differences in postprandial iAUC responses between the OB and T2D groups versus the HC group (Table 6).

Compared to the HC group, the OB group exhibited significantly higher iAUC values for glucose (β = +4937.6, 95% CI 3752.2 to 6123.0, *p* < 0.001), insulin (β = +1798.6, 95% CI 668.7 to 2928.5, *p* = 0.002) and C-peptide (β = +101.4, 95% CI 5.7 to 197.2, *p* = 0.038). The T2D group had significantly elevated iAUC values for glucose (β = +1293.4, 95% CI 104.7 to 2482.0, *p* = 0.033) compared to the HC group. No significant differences between groups were observed in the iAUCs for GLP-1, leptin, IL-6, ghrelin, or PYY.

### 3.6. Bread × Group Interaction Effects

Exploratory LMMs incorporated bread type × group interaction terms to investigate whether the postprandial response to pelemir-enriched bread differed by metabolic status. While no statistically significant interactions were detected after adjustment for multiple comparisons, a trend toward a greater increase in iAUC for GLP-1 with pelemir-enriched bread was observed in the OB group compared to the HC group (*p* for interaction = 0.085).

### 3.7. VAS Findings (Subjective Appetite Ratings)

Postprandial subjective appetite responses, as assessed by VAS-derived iAUC values, are presented in Table 7. No statistically significant differences were observed between pelemir-enriched and regular breads in terms of hunger (*p* = 0.576), fullness (*p* = 0.683), or desire to eat (*p* = 0.285). However, the prospective food consumption scores were significantly lower after pelemir-enriched bread compared to regular bread (4950.0 [3015.0–6300.0] vs. 5475.0 [3832.5–7470.0], *p* = 0.050), suggesting a modest reduction in anticipated food intake.

## 4. Discussion

This non-randomized, two-phase study evaluated the acute postprandial metabolic effects of pelemir-enriched bread compared to regular white bread in individuals with and without metabolic disturbances. In our study, consumption of pelemir-enriched bread was associated with higher postprandial iAUC values for insulin, C-peptide, and GLP-1 compared to regular bread, without a concomitant change in iAUC for glucose. This hormonal profile could be interpreted in different ways. On the one hand, the simultaneous increase in insulin and GLP-1 may reflect an improved early-phase β-cell response and incretin-mediated stimulation, potentially improving postprandial glucose clearance. However, the requirement for greater insulin secretion in the absence of a glucose change could also suggest reduced insulin efficiency, particularly in insulin-resistant states. Given the acute nature of the intervention and the lack of direct dynamic measures of insulin sensitivity, our findings should be considered indicative of altered postprandial endocrine dynamics rather than definitive improvements in metabolic control. Future studies incorporating gold standard methods, such as euglycemic–hyperinsulinemic clamps or frequently sampled oral glucose tolerance tests, are needed to clarify whether this pattern reflects beneficial β-cell stimulation, compensatory hyperinsulinemia, or both. This dissociation between glucose and insulin/GLP-1 responses may also suggest a possible modification in glycemic regulation through nonnutrient-mediated mechanisms, such as gut-derived signaling or polyphenol-induced incretin modulation [18,19].

Importantly, the metabolic effect of pelemir-enriched bread cannot be explained by macronutrient composition alone. As detailed in Table 1, pelemir-enriched bread actually contained a slightly lower fiber content than the control bread, which may be due to dilution of wheat bran in the composite flour or natural variability in formulation. Since the primary target of pelemir supplementation was polyphenolic bioactivity rather than fiber enrichment, this difference in fiber content was not considered critical to the study results. Furthermore, the protein and fat contents were also comparable. These findings support the hypothesis that the bioactive compounds inherent in pelemir, such as polyphenols and phytochemicals, may underlie the observed effects [20]. While our study did not quantify the total phenolic content, prior analyses of related ancestral wheats have shown that these grains are rich in antioxidant and anti-inflammatory compounds that can influence glucose metabolism through the incretin or intestinal–brain axis pathways [21].

One potential concern is the low amount of added pelemir (0.3% of the wheat flour) used in this study. This dose reflects industrial limitations, as higher levels (0.5%) resulted in an unpalatable bitter taste, as reported by food engineers from the partnering bakery, and consequently was considered unacceptable for consumer use. Thus, the dose used in this study reflects the highest level of organoleptically tolerable and commercially feasible addition of pelemir. Therefore, our findings are relevant for real-world product development and diet strategies.

From a statistical point of view, we strengthened our analysis by applying LMMs with bread × group interaction terms, nonparametric testing for skewed distributions, and robust within-subject comparisons. Although interaction effects did not reach statistical significance, the trend toward an enhanced GLP-1 response in the HCgroup suggests a possible group-specific sensitivity to pelemir, meriting further investigation.

Comparisons between groups highlighted expected differences in postprandial dynamics. Both OB and T2D groups exhibited significantly elevated iAUC for glucose and insulin compared to the HC group. These findings are consistent with the established literature on postprandial dysregulation in metabolic diseases [22,23]. However, the lack of significant differences in GLP-1 and PYY responses may reflect blunted incretin sensitivity rather than absolute deficiency or may be due to pharmacological modulation in the T2D group [24].

Although IL-6 is generally regarded as a systemic inflammation marker that responds over longer periods, previous studies have shown that even a single high-fat or carbohydrate meal can increase postprandial IL-6 concentrations acutely within a few hours. Therefore, IL-6 was included as a postprandial parameter to investigate whether pelemir-enriched bread, rich in polyphenols and bioactive compounds, could influence early inflammation signaling compared to regular bread [25,26]. Unfortunately, high interindividual variability observed in IL-6, leptin, and ghrelin responses across all groups underscores the complexity of postprandial hormonal regulation. While trends were observed for elevated inflammatory markers in metabolically affected individuals, none reached statistical significance, possibly due to our modest sample size as well as low amount of added pelemir. This variability supports the need for stratified analyses and highlights the importance of expanding future research to include inflammatory phenotyping [27].

In addition to glycemic and hormonal responses, we evaluated subjective postprandial appetite sensations using VAS. While no significant differences were found between pelemir-enriched and regular breads in hunger, fullness, or desire to eat, the prospective food consumption scores were significantly lower after consumption of pelemir (*p* = 0.050). This borderline significant finding may suggest that pelemir-enriched bread modestly reduced participants’ anticipation of future food intake, potentially reflecting subtle appetite-modulating effects. Although the observed differences did not extend to immediate satiety signals, these results align with previous studies indicating that polyphenol-rich or bitter-tasting functional foods may influence hedonic and anticipatory components of appetite through sensory or gut–brain mechanisms [16,28]. Given the mild sensory bitterness of pelemir-enriched bread reported during production trials, this result is biologically plausible, though it requires confirmation in larger studies.

### Strengths and Limitations

This study presents several notable strengths. The use of a real-life food product, white bread enriched with a commercially feasible dose of pelemir, improves ecological validity and applicability to daily nutrition. Furthermore, the inclusion of individuals across the metabolic spectrum (healthy, obese, and type 2 diabetes) allowed for wider generalizability and group-specific insights.

Nevertheless, certain limitations should be acknowledged. The most important and major limitation of our study is the lack of randomization both to the order of test meals and between groups in terms of variables such as age, sex, and BMI due to the difficulties in finding participants who both met the inclusion criteria and were available to participate in the study procedure. Second, the short-term nature of the intervention precludes conclusions regarding long-term metabolic adaptations or chronic health outcomes. Third, while the pelemir content was sufficient to produce measurable effects, its low amount may limit the magnitude of impact expected to be observed. Fourth, the absence of detailed compositional profiling of pelemir flour, including polyphenol and phytochemical content, restricts mechanistic interpretation. The possible impact of antihyperglycemic medications used by the patients on the results can be considered as an additional limitation. Finally, the modest sample size may have limited statistical power, particularly for interaction analyses and low-variance hormonal markers.

## 5. Conclusions

This study demonstrates that even a minimal addition of pelemir seed flour (0.3%) to white bread can acutely influence postprandial insulin and GLP-1 responses without adversely affecting glycemia. These effects were observed in healthy individuals, as well as obese and type 2 diabetes patients, suggesting that pelemir may represent a feasible functional ingredient to modulate postprandial endocrine responses. Although the underlying mechanisms probably involve polyphenolic and nonnutritive components, more research is needed to confirm these findings, including exploration for dose–response relationships, and to evaluate long-term clinical implications.

## Figures and Tables

**Table 1:** Comparative nutritional composition and ingredient profile of regular and pelemir-enriched breads *.

Component	Regular Bread	Pelemir-Enriched Bread
Bread weight (g)	95	100
Carbohydrates (g)	50	50
Energy (kcal)	291.5	285.4
Dietary fibers (g)	3.23	3.12
Dietary fiber (%)	3.4	3.12
Fats (total, g)	2.14	2.64
Fat (total, %)	2.25	2.64
Saturated fats (g)	0.49	0.8
Saturated fat (%)	0.52	0.8
Proteins (g)	8.74	10.1
Protein (%)	9.2	10.1
Pelemir flour (%)	0	0.3
NaCl (g)	0.68	0.7
NaCl (%)	0.71	0.7

Values are based on the actual serving sizes used in the study (95 g for regular bread and 100 g for pelemir-enriched bread, both containing 50 g carbohydrates). Percentages refer to the proportion of the total bread weight. Nutrient values were obtained from standardized manufacturer data. * As per 50 g of carbohydrate equivalent portion.

**Table 2:** General characteristics of the study groups.

Characteristic	HC (*n* = 20)	OB (*n* = 20)	T2D (*n* = 20)	*p*-Value
Women/Men (*n*)	18/2	7/13	13/7	0.001
Age (year)	38.0 (32.0–45.8)	39.5 (33.5–44.3)	52.7 (46.0–59.0)	<0.001
BMI (kg/m^2^)	22.5 (20.9–23.7)	35.1 (31.5–38.2)	31.5 (25.8–37.6)	<0.001
Weight (kg)	61.6 (57.3–65.6)	99.4 (90.6–109.1)	80.1 (69.5–90.1)	<0.001
Height (cm)	165.8 (160.3–171.3)	168.1 (162.0–174.2)	162.7 (156.0–169.3)	0.084
Fat mass (%)	26.4 (21.0–30.7)	34.3 (30.1–38.6)	32.8 (29.5–36.0)	0.002
Fat mass (kg)	16.5 (12.9–19.8)	34.1 (27.9–38.9)	26.3 (21.8–29.9)	<0.001
FFM (kg)	45.2 (42.4–47.9)	65.2 (57.2–70.1)	53.8 (47.3–59.6)	<0.001

Abbreviations: HC, healthy control group; OB, group with obesity; T2D, group with type 2 diabetes; BMI, body mass index; FFM, fat-free mass; IQR, interquartile range. Data are given as median (IQR). The *p*-values reflect comparisons between groups based on the Kruskal–Wallis test.

**Table 3:** Comparison of fasting biochemical parameters before the two test meals (regular vs. pelemir-enriched breads).

Parameter	Regular Bread (Min–Max)	Pelemir-Enriched Bread (Min–Max)	*p*-Value
Glucose * (mg/dL)	81.5 (60.0–191.0)	79.0 (45.0–219.0)	0.748
Insulin ** (µU/mL)	7.7 (0.5–25.6)	7.7 (0.8–32.3)	0.994
C-Peptide * (ng/mL)	2.0 (0.6–4.1)	2.1 (0.7–7.1)	0.051
Leptin ** (pg/mL)	1347.0 (66.0–5877.0)	1005.0 (53.0–5251.0)	0.063
PYY * (pg/mL)	63.8 (1.5–591.8)	58.0 (6.4–342.0)	0.485
GLP-1 * (pg/mL)	11.9 (3.1–842.9)	11.7 (3.1–1684.0)	0.460
Triglycerides ** (mg/dL)	91 (37.0–448.0)	90 (39.0–327.0)	0.756
IL-6 * (pg/mL)	2.6 (0.4–81.6)	2.9 (0.4–126.3)	0.384
Ghrelin ** (ng/mL)	0.7 (0.2–9.3)	0.8 (0.1–9.0)	0.197

Abbreviations: HC, healthy control group; OB, group with obesity; T2D, group with type 2 diabetes; PYY, peptide YY; GLP-1, glucagon-like peptide-1; IL-6, interleukin-6. Data are presented as median (minimum–maximum). Comparisons between test meals were made using the Wilcoxon signed-rank test (*) or paired *t*-test (**), depending on distributional characteristics.

**Table 4:** Group-specific comparison of postprandial incremental areas under the curves between test meals (regular vs. pelemir-enriched breads).

Parameter	Group	Regular Bread (Min–Max)	Pelemir-Enriched Bread (Min–Max)	*p*-Value
Glucose (mg.min/dL)	HC	179.0 (133.8–220.8)	177.5 (143.0–209.8)	0.984
	OB	325.2 (209.2–490.0)	334.5 (214.2–615.5)	0.679
	T2D	196.8 (140.5–344.2)	200.5 (136.2–301.5)	0.347
Insulin (µU.min/mL)	HC	38.1 (17.6–62.8)	38.1 (15.9–95.7)	0.738
	OB	63.4 (26.6–208.4)	82.9 (19.7–192.0)	0.096
	T2D	49.4 (11.2–152.7)	56.2 (11.5–208.8)	0.284
C-Peptide (ng.min/mL)	HC	7.5 (4.3–9.5)	8.0 (3.8–16.1)	0.073
	OB	11.1 (5.1–19.5)	10.8 (6.0–24.8)	0.049
	T2D	9.4 (3.2–14.9)	9.8 (3.9–21.7)	0.099
Leptin (pg.min/mL)	HC	725.6 (110.6–4698.8)	772.2 (110.0–4987.0)	0.648
	OB	2448.8 (239.5–10,366.2)	2842.5 (131.5–8966.5)	0.922
	T2D	3527.0 (567.8–10,349.0)	3018.8 (322.8–10,502.0)	0.246
PYY (pg.min/mL)	HC	128.6 (24.3–908.1)	140.5 (27.1–930.6)	0.083
	OB	115.6 (47.9–304.9)	107.1 (19.5–333.6)	0.651
	T2D	114.0 (7.1–311.8)	100.1 (23.6–234.3)	0.119
GLP-1 (pg.min/mL)	HC	197.7 (7.4–994.1)	414.6 (14.6–1801.6)	0.022
	OB	22.4 (20.0–628.8)	22.0 (10.3–570.4)	0.241
	T2D	22.4 (6.8–1362.3)	21.8 (11.4–1706.6)	0.442
Triglyceride (mg.min/dL)	HC	130 (72.2–297.2)	122 (64.2–268.8)	0.984
	OB	338 (131.8–786.2)	289 (116.5–639.2)	0.798
	T2D	229 (98.2–681.8)	216 (97.5–648.5)	0.610
IL-6 (pg.min/mL)	HC	6.6 (1.1–64.9)	17.2 (0.8–83.0)	0.070
	OB	4.4 (0.8–36.3)	3.6 (0.8–87.6)	0.156
	T2D	4.1 (1.3–99.0)	5.0 (1.8–409.6)	0.347
Ghrelin (ng.min/mL)	HC	4.4 (0.7–18.2)	4.8 (0.4–15.5)	0.729
	OB	1.1 (0.5–4.2)	1.6 (0.4–8.7)	0.123
	T2D	1.0 (0.3–5.1)	1.3 (0.4–7.2)	0.304

Abbreviations: HC, healthy control group; OB, group with obesity; T2D, group with type 2 diabetes; PYY, peptide YY; GLP-1, glucagon-like peptide-1; IL-6, interleukin-6. Data are shown as median (minimum–maximum) for each metabolic group. Within-group comparisons were made using the Wilcoxon signed-rank test.

**Table 5:** Effect of bread type (pelemir-enriched vs. regular) on incremental areas under the curve for postprandial responses *.

Parameter	Estimated Difference (P vs. R)	95% CI	*p*-Value
Glucose (mg.min/dL)	+161.2	−360.6 to +683.0	0.545
Insulin (µU.min/mL)	+555.6	+110.2 to +1001.0	0.014
C-Peptide (ng.min/mL)	+35.9	+0.7 to +71.1	0.046
Leptin (pg.min/mL)	+232.0	−5441.5 to +5905.5	0.936
PYY (pg.min/mL)	+313.9	−326.2 to +953.9	0.337
GLP-1 (pg.min/mL)	+1234.8	+60.3 to +2409.2	0.039
Triglyceride (mg.min/dL)	+309.6	−89.3 to +708.5	0.128
IL-6 (pg.min/mL)	+352.9	−27.8 to +733.7	0.069
Ghrelin (ng.min/mL)	+5.7	−6.4 to +17.8	0.356

Abbreviations: P, pelemir-enriched; R, regular; CI, confidence interval; PYY, peptide YY; GLP-1, glucagon-like peptide-1; IL-6, interleukin-6. * The difference represents adjusted marginal mean differences from linear mixed-effects models, adjusted for group and participant’s ID as a random intercept. These estimates are not direct subtractions of median values and may differ in magnitude due to model adjustment and pooling of data across all participants.

**Table 6:** Comparison of incremental areas under the curve for postprandial responses between obese and type 2 diabetes groups vs. healthy control group *.

Parameter	Comparisons	Difference (OB or T2D vs. HC)	95% CI	*p*-Value
Glucose (mg.min/dL)	OB vs. HC	+4937.6	+3752.2 to +6123.0	<0.001
	T2D vs. HC	+1293.4	+104.7 to +2482.0	0.033
Insulin (µU.min/mL)	OB vs. HC	+1798.6	+668.7 to +2928.5	0.002
	T2D vs. HC	+1049.4	−83.1 to +2182.0	0.069
C-Peptide (ng.min/mL)	OB vs. HC	+101.4	+5.7 to +197.2	0.038
	T2D vs. HC	+55.8	−39.1 to +150.7	0.244
Leptin (pg.min/mL)	OB vs. HC	+2472.8	−5593.1 to +10,538.6	0.548
	T2D vs. HC	+4771.2	−3340.6 to +12,883.1	0.249
PYY (pg.min/mL)	OB vs. HC	−269.8	−1080.6 to +541.0	0.514
	T2D vs. HC	−699.1	−1514.7 to +116.5	0.093
GLP-1 (pg.min/mL)	OB vs. HC	−1662.1	−3374.1 to +49.9	0.057
	T2D vs. HC	−1021.0	−2731.5 to +689.6	0.236
Triglyceride (mg.min/dL)	OB vs. HC	+285	−156.5 to +727.3	0.198
	T2D vs. HC	+436	−4.1 to +875.6	0.052
IL-6 (pg.min/mL)	OB vs. HC	+329.9	−53.4 to +713.3	0.091
	T2D vs. HC	+296.0	−90.0 to +682.0	0.132
Ghrelin (ng.min/mL)	OB vs. HC	+2.4	−9.0 to +13.8	0.673
	T2D vs. HC	−6.6	−18.1 to +4.9	0.247

Abbreviations: HC, healthy control group; OB, group with obesity; T2D, group with type 2 diabetes; CI, confidence interval; PYY, peptide YY; GLP-1, glucagon-like peptide-1; IL-6, interleukin-6. * The difference represents adjusted marginal mean differences from linear mixed-effects models, adjusted for group and participant’s ID as a random intercept. These estimates are not direct subtractions of median values and may differ in magnitude due to model adjustment and pooling of data across all participants.

**Table 7:** Comparison of incremental areas under the curve for subjective appetite ratings Between regular vs. pelemir-enriched breads.

Parameter	Regular Bread	Pelemir-Enriched Bread	*p*-Value
Hunger (mm.min)	3840.0 (2445.0–5887.5)	4350.0 (3030.0–5640.0)	0.576
Fullness (mm.min)	6735.0 (5276.2–8152.5)	6877.5 (5557.5–8512.5)	0.683
Desire to Eat (mm.min)	5602.5 (3592.5–7383.8)	5362.5 (3221.2–6828.8)	0.285
Prospective Food Consumption (mm.min)	5475.0 (3832.5–7470.0)	4950.0 (3015.0–6300.0)	0.050

Values are presented as median [interquartile range]. iAUC: incremental area under the curve. Statistical comparisons were made using the Wilcoxon signed-rank test. *p*-values < 0.05 were considered statistically significant.

## Data Availability

The data presented in this study are available upon request from the corresponding author.
